# Variable Ventilation Is Equally Effective as Conventional Pressure Control Ventilation for Optimizing Lung Function in a Rabbit Model of ARDS

**DOI:** 10.3389/fphys.2019.00803

**Published:** 2019-06-26

**Authors:** Gergely H. Fodor, Sam Bayat, Gergely Albu, Na Lin, Aurélie Baudat, Judit Danis, Ferenc Peták, Walid Habre

**Affiliations:** ^1^Unit for Anesthesiological Investigations, Geneva University Hospitals – University of Geneva, Geneva, Switzerland; ^2^Inserm UA7 STROBE Laboratory, Department of Clinical Physiology, Sleep and Exercise, Grenoble University Hospital, Grenoble, France; ^3^Department of Anesthesiology, Beijing Tongren Hospital, Capital Medical University, Beijing, China; ^4^MTA-SZTE Dermatological Research Group, University of Szeged, Szeged, Hungary; ^5^Department of Medical Physics and Informatics, University of Szeged, Szeged, Hungary

**Keywords:** ventilator-induced lung injury, variable ventilation, ARDS, respiratory mechanics, gas exchange, PEEP, protective ventilation

## Abstract

**Background:**

Introducing mathematically derived variability (MVV) into the otherwise monotonous conventional mechanical ventilation has been suggested to improve lung recruitment and gas exchange. Although the application of a ventilation pattern based on variations in physiological breathing (PVV) is beneficial for healthy lungs, its value in the presence of acute respiratory distress syndrome (ARDS) has not been characterized. We therefore aimed at comparing conventional pressure-controlled ventilation with (PCS) or without regular sighs (PCV) to MVV and PVV at two levels of positive end-expiratory pressure (PEEP) in a model of severe ARDS.

**Methods:**

Anesthetised rabbits (*n* = 54) were mechanically ventilated and severe ARDS (PaO_2_/FiO_2_ ≤ 150 mmHg) was induced by combining whole lung lavage, i.v. endotoxin and injurious ventilation. Rabbits were then randomly assigned to be ventilated with PVV, MVV, PCV, or PCS for 5 h while maintaining either 6 or 9 cmH_2_O PEEP. Ventilation parameters, blood gas indices and respiratory mechanics (tissue damping, G, and elastance, H) were recorded hourly. Serum cytokine levels were assessed with ELISA and lung histology was analyzed.

**Results:**

Although no progression of lung injury was observed after 5 h of ventilation at PEEP 6 cmH_2_O with PVV and PCV, values for G (58.8 ± 71.1[half-width of 95% CI]% and 40.8 ± 39.0%, respectively), H (54.5 ± 57.2%, 50.7 ± 28.3%), partial pressure of carbon-dioxide (PaCO_2_, 43.9 ± 23.8%, 46.2 ± 35.4%) and pH (−4.6 ± 3.3%, −4.6 ± 2.2%) worsened with PCS and MVV. Regardless of ventilation pattern, application of a higher PEEP improved lung function and precluded progression of lung injury and inflammation. Histology lung injury scores were elevated in all groups with no difference between groups at either PEEP level.

**Conclusion:**

At moderate PEEP, variable ventilation based on a pre-recorded physiological breathing pattern protected against progression of lung injury equally to the conventional pressure-controlled mode, whereas mathematical variability or application of regular sighs caused worsening in lung mechanics. This outcome may be related to the excessive increases in peak inspiratory pressure with the latter ventilation modes. However, a greater benefit on respiratory mechanics and gas exchange could be obtained by elevating PEEP, compared to the ventilation mode in severe ARDS.

## Introduction

Positive-pressure mechanical ventilation is an essential life-support measure to maintain gas exchange in patients with acute respiratory failure or undergoing general anesthesia. However, its prolonged application may lead to ventilation-induced lung injury (VILI). Efforts have been made in the last decades to suggest various ventilation strategies to prevent the occurrence of VILI. The most commonly promoted preventive strategy is based on the protective ventilation concept with a restricted tidal volume (V_T_) associated with high positive end-expiratory pressure (PEEP) levels and lung recruitment maneuvres (Reiss et al., 2011; Bayat et al., 2013; Sahetya et al., 2017; Fan et al., 2018). However, the individual roles of V_T_, PEEP, plateau pressure, and driving pressure (defined as plateau pressure – PEEP) are highly debated (Ochiai, 2015; Guerin et al., 2016). Moreover, low V_T_ ventilation can also promote lung injury due to repeated recruitment of collapsed airspaces or atelectrauma (Muscedere et al., 1994; Gattinoni et al., 2006; Retamal et al., 2015). Therefore, the development of alternative ventilation modalities to avoid the adverse effects of monotonous positive-pressure mechanical ventilation while providing adequate gas exchange, remains crucial (Cordioli et al., 2013).

Among novel ventilation modalities, application of a variable V_T_ and/or frequency has been proposed to enhance alveolar recruitment thereby optimizing lung aeration and gas exchange (Suki et al., 1998; Vassilakopoulos and Zakynthinos, 2008). The variability in V_T_ in these previous studies was defined by various statistical distributions, including Gaussian normal (Kiss et al., 2016; Wierzchon et al., 2017; Camilo et al., 2018; Guldner et al., 2018; Spieth et al., 2018) or skewed probability density distributions (Thammanomai et al., 2008; Bartolak-Suki et al., 2017). In an attempt to extend this approach, we recently developed a variable ventilation modality based on a pre-recorded physiological breathing pattern (Walesa et al., 2018). This physiologically variable ventilation (PVV) has been shown to be beneficial for providing optimal gas exchange and minimizing the deleterious consequences of prolonged mechanical ventilation in animals with healthy lungs. However, the added potential benefit of PVV to the already established advantage of high PEEP levels in the presence of acute respiratory distress syndrome (ARDS) is not known. In addition, the value of introducing physiological variability into mechanical ventilation has not been compared to the application of mathematically derived V_T_ variability or conventional monotonous ventilation modes.

Therefore, we aimed at characterizing the value of variable ventilation based on a physiological breathing pattern PVV in an experimental model of ARDS and comparing the respiratory mechanical and gas exchange parameters to those obtained with a mathematically derived variable ventilation (MVV), a conventional pressure-controlled mode (PCV), or a pressure-controlled mode with regular sighs (PCS). Since management of ARDS invariably requires elevating PEEP, we also compared these ventilation modes under moderate (6 cmH_2_O, MP) and high (9 cmH_2_O, HP) PEEP levels.

## Materials And Methods

### Ethics Statement

The experimental protocol was approved by the Experimental Ethics Committee of the University of Geneva and the Animal Welfare Committee of the Canton of Geneva, Switzerland (No. GE/94/15, August 27, 2015). All procedures were performed according to the current animal protection laws of Switzerland (LPA, RS455) and reported in compliance with ARRIVE guidelines.

### Experimental Animals

Male and female adult white New Zealand rabbits were purchased from the farm of University of Geneva (Arare, Geneva, Switzerland) for the present study (mean weight 3.13 kg, ranging from 2.67–3.99 kg). Animals had access to food and water *ad libitum* before the experiment and welfare of the animals was ensured according to the current animal protection laws of Switzerland.

### Study Design

**FIGURE 1:**
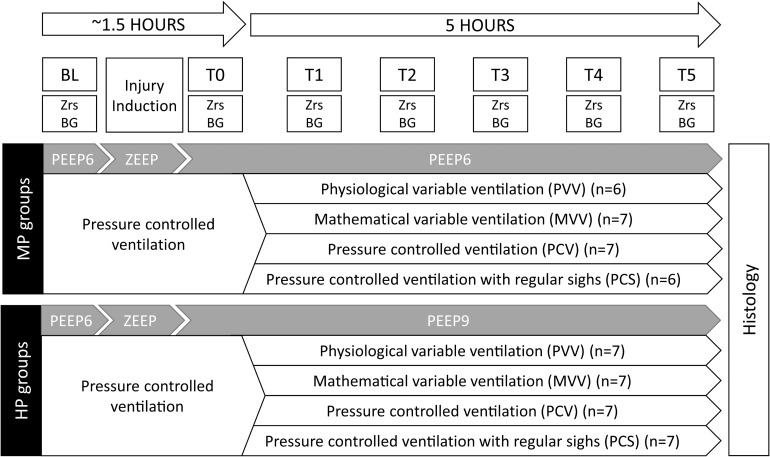
Overview of the experimental protocol. BL, baseline; T0, measurement point following lung injury but preceding the application of various ventilation modes; T1–T5, measurement points at the end of each 5 h of ventilation with an experimental pattern; Zrs, respiratory impedance data collection; BG, blood gas measurement; PEEP, positive end-expiratory pressure; ZEEP, zero end-expiratory pressure; MP, moderate PEEP; HP, high PEEP.

The study protocol is outlined in Figure 1. Ventilation was initiated in supine position with a PEEP of 6 cmH_2_O using pressure-controlled mode in all groups. Following animal preparation, anesthesia and surgery (detailed below), lung volume history was standardized by applying 2 deep inflations (30 cmH_2_O peak pressure maintained for 5 s each). After 5 min, when the vital parameters stabilized, baseline parameters (BL) were obtained: arterial and venous blood gas samples were taken and a set of respiratory input impedance spectra (Zrs) was recorded.

A model of severe ARDS characterized by a ratio of arterial oxygen partial pressure and fraction of inspired oxygen (PaO_2_/FiO_2_) of approximately 100 mmHg, according to the Berlin definition (ARDS Definition Task Force et al., 2012), was then induced with a multi-hit model including the combination of a single intravenous injection of 300 μg/kg lipopolysaccharide (LPS, from *Escherichia coli* O111:B4, Sigma, Saint Louis, Missouri, United States), repeated whole-lung lavage (instilling and removing 5 × 60 ml 37°C normal saline through the endotracheal tube) and injurious ventilation (0 cmH_2_O end-expiratory pressure, ZEEP, *V*_T_ = 10 ml/kg and FiO_2_ = 1) for 20–30 min. Lavage fluid was recovered as fully as possible without the application of suction. Following this period, the animals were randomized to be ventilated with a PEEP of either 6 or 9 cmH_2_O and FiO_2_ was decreased to 0.9. An arterial blood gas sample was taken. If the target PaO_2_/FiO_2_ ratio had not been achieved, injurious ventilation was repeated for further 15-min periods. When the target range of PaO_2_/FiO_2_ was reached, a recruitment maneuvre was performed (two deep inflations of 30 cmH_2_O peak pressure maintained for 5 s each). Then, following a 5-min stabilization period another set of Zrs and blood gas data was collected (T0).

The animals were further randomized following the data collection at T0, to be ventilated with either conventional pressure-controlled mode (PCV, regular respiratory rate and V_T_), pressure-controlled mode with regular sighs (PCS, 2 sighs per minute, defined as a normal-length breath with a peak inspiratory pressure of 35 cmH_2_O, otherwise regular respiratory rate and V_T_), variable ventilation based on pre-recorded, physiological breathing signal (PVV) (Walesa et al., 2018) or variable ventilation based on a mathematical probability distribution of V_T_ designed to optimize lung recruitment following injury (mathematically derived variability, MVV) (Thammanomai et al., 2008). The applied ventilation patterns are demonstrated in Supplementary Figure S1. These ventilation modes were maintained for 5 h, with hourly data collection (T1–T5). Average and median V_T_ were checked hourly and ventilatory pressures were adjusted to achieve an average V_T_ of 6 ml/kg. During this 5-h period, animals in all groups were ventilated with the same FiO_2_ of 0.9, and an inspiratory / expiratory ratio (I:E) of 1:2 was applied. At the end of the protocol, animals were euthanized, and the lungs were extracted for histological analyses.

### Allocating Animals to Experimental Groups

A block randomization procedure was used to assign the animals into one of the eight experimental groups. The website randomizer.org was used to generate the blocks.

### Experimental Outcomes

The primary outcomes of the present study were defined as respiratory mechanical parameters (Raw, G and H) and arterial blood gas parameters (lactate, pH, PaO_2_, and PaCO_2_). Secondary outcomes were haemodynamic parameters, serum cytokine levels and lung injury indices obtained from lung histology.

### Experimental Procedures

All experiments were carried out at the laboratory of the Unit for Anesthesiological Investigations, University Hospitals of Geneva and University of Geneva.

#### Anesthesia and Surgical Preparation

All experimental animals were sedated with an intramuscular injection of xylazine (5.0 mg/kg). A 22 G catheter was inserted into a marginal ear vein and a continuous intravenous infusion of propofol (15–20 mg/kg/h) and fentanyl (5 μg/kg/h) was administered to induce and maintain general anesthesia. Tracheostomy was carried out after infiltrating the surgical site with lidocaine and a 3.0 mm uncuffed tube was introduced into the trachea. A V_T_ of 7 ml/kg and a respiratory rate to achieve normocapnia (5.5–6% end-tidal CO_2_, approximately 40 breaths per minute at baseline) was used to initiate and maintain mechanical ventilation. An FiO_2_ of 0.4 and a PEEP of 6 cmH_2_O were applied using the pressure-controlled mode of a pediatric ventilator (Servo-i, Maquet Critical Care, Solna, Sweden). When proper depth of anesthesia was reached, a continuous infusion of atracurium (0.6 mg/kg/h) was initiated to ensure neuromuscular blockade. A continuous infusion of lactated Ringer’s solution (4 ml/kg/h) was also administered to maintain fluid balance.

A 22 G catheter was placed in the left femoral artery (Abbocath, Abbot Medical, Baar/Zug, Switzerland) for invasive blood pressure monitoring and intermittent blood withdrawal for arterial blood gas analysis. A 16 G catheter was introduced into the right jugular vein (Arrow, Teleflex Medical Europe, Westmeath, Ireland) and was used to take central venous blood gas samples. Subcutaneous needle electrodes were used to monitor electrocardiogram. A thermostatic heating pad (Harvard Apparatus, South Natick, MA, United States) was placed under the animals and a rectal thermometer probe was also used to keep internal body temperature within the range around 38–39°C. Tracheal pressure and airflow, arterial pressure and electrocardiogram were digitized (sampling rate 1 kHz) and continuously recorded (ADInstruments, Powerlab model 8/35 and LabChart 7, Dunedin, New Zealand). Mean arterial pressure (MAP) and heart rate (HR) were calculated from the recorded traces. Animals were euthanized with an overdose of intravenous thiopental (100 mg/kg, 2 ml/kg) while maintaining general anesthesia.

#### Measurement of Respiratory Mechanics

The wave-tube method of the forced oscillation technique was used to assess the airway and respiratory tissue mechanical parameters, as detailed previously (Bayat et al., 2009). Briefly, a small-amplitude (1 cmH_2_O peak-to-peak) pseudorandom, low-frequency (0.5–20.75 Hz) forcing signal was generated by a loudspeaker-in-box system. This forcing signal was introduced through a polyethylene wave-tube (100 cm length, 0.375 cm internal diameter) into the tracheal cannula during short (8 s) end-expiratory apneas. The wave-tube and the loudspeaker chamber were pressurized to the level of PEEP to avoid changes in the mean airway pressure during the recordings. Lateral pressures were measured at the loudspeaker (P_1_) and the tracheal end (P_2_) of the wave-tube using miniature pressure transducers (ICS 33NA00D, Milpitas, CA, United States). The pressure signals were low-pass filtered at 25 Hz corner frequency and digitized at 256 Hz using an analog-digital converter board (USB-6211, National Instruments, Austin, TX, United States). Zrs was derived from the fast Fourier transform of the pressure transfer function (P_1_/P_2_) (Franken et al., 1981). Three to four comparable 8 s recordings were taken during each time point and these Zrs spectra were ensemble averaged for further analysis.

A well-validated model with airway tissue compartments was fitted to the Zrs spectra using a global optimization method. This model comprises an airway resistance (Raw) and airway inertance (Iaw) in series with a constant-phase tissue compartment including tissue damping (G) and tissue elastance (H) (Hantos et al., 1992). As previously established (Petak et al., 1997), Raw reflects primarily the flow resistance of the central conducting airways, Iaw is related to the cyclic acceleration and deceleration of the gas content of the central airways, G describes the energy loss within the respiratory tissues (resistance) whereas H characterizes the energy storage capacity of the respiratory tissues (elastance). The impedance of the endotracheal tube and the connecting circuit was measured, and these instrumental components were all subtracted from the Zrs spectra prior to model fitting.

#### Blood Gas Analyses

A point-of-care blood gas analyser (i-Stat, Abbott Laboratories, Chicago, IL, United States) was used to determine PaO_2_ and PaCO_2_, pH and lactate concentration from arterial and venous blood samples.

#### Application of Variable Ventilation

The ventilator was driven by a computer to generate variable ventilation patterns in a looped manner using custom-made software. The software controlled the timing and amplitudes of the respiratory pressure peaks, aiming for a median V_*T*_ of 6 ml/kg, which was checked hourly and adjusted if necessary. The physiological breathing pattern used in the PVV groups was recorded in healthy animals during a previous experiment (Walesa et al., 2018) with a nasogastric electrode to record the electrical activity of the diaphragm. The variable pattern applied in the MVV groups was generated according to the previously reported data by Thammanomai et al. (2008). The regular sighs applied in PCS groups were set to occur every 30 s. The ventilatory patterns, as well as their characteristics are presented in the online data supplement.

#### Immunology

The enzyme-linked immunosorbent assay was performed on blood serum samples obtained at the beginning (BL) and the end (T5) of the experimental protocol to assess the presence of inflammatory cytokines TNF-α (MyBiosource MBS2021700, San Diego, CA, United States), IL-1β and IL-8 (Raybiotech Norcross, GA, United States). Measurements were performed according to the manufacturer’s instructions.

#### Lung Histology

Four percent formaldehyde was filled into the left lung at a hydrostatic pressure of 20 cmH_2_O. Apical, middle and basal lobe regions were excised and fixed before embedding them in paraffin. Lung tissue sections (5 μm) were stained with hematoxylin and eosin. An expert technician blinded to group allocations performed the analysis in accordance with American Thoracic Society guidelines (Matute-Bello et al., 2011). Lung injury was quantified by calculating a lung injury score, which includes the presence of neutrophils in the alveolar and interstitial spaces, hyaline membranes, proteinaceous debris filling the airspaces and alveolar septal thickening in a weighted manner.

### Sample Size

Sixty-four rabbits were randomized into one of eight experimental groups. The sample sizes were estimated based on our previous data for respiratory tissue elastance (H) (Doras et al., 2015) as the main outcome variable to detect 20% between-group differences in the injured lung; assuming a coefficient of variation of 10%, a statistical power of 0.8 and 2-sided alpha error of 0.05. The estimation resulted in a required sample size of seven rabbits per group. Considering the potential drop-out rate of about 15%, we attempted to include 8 animals in each experimental group.

### Statistical Methods

Data are presented as mean ± half-width of 95% confidence interval. Three-way repeated measure analyses of variances (ANOVA) using linear mixed model fits by a restricted maximum likelihood (REML) method were carried out on respiratory mechanical, hemodynamic, blood gas parameters and cytokine levels. Normality of the data was assessed for each variable by the use of Shapiro-Wilk test. In case of a failed normality test, the variable was log-transformed. To account for the multiple three-way repeated measures comparisons and to reduce the probability of Type I error, Dunnett’s *post hoc* tests using reference levels were carried out on these parameters to assess the time effects (with T0 as reference point), the effects of PEEP (6 vs. 9 cmH_2_O, with 6 cmH_2_O as the reference level) and ventilation pattern (PVV as the reference mode). Histology parameters were analyzed using two-way ANOVA with Holm-Sidak *post hoc* tests using PEEP and ventilation mode as between-group variables. To take the effects of the multiple repeated measures comparisons into account, the validity of the multiple three-way ANOVA analyses was assessed using a principle component analysis on the primary outcome variables. The statistical tests were performed within the *R* environment with the *lme4* (Bates et al., 2015), *lsmeans* (Lenth, 2016) and *stats* packages and SigmaPlot (Version 13, Systat Software, Inc., Chicago, IL, United States). The statistical tests were performed with a significance level of *p* < 0.05, and all *p*-values were two-sided.

## Results

Evolution of the ventilation parameters are presented in the online data supplement (Supplementary Figures S2–S6). Ten rabbits were excluded from the analysis due to either the development of pneumothorax (*n* = 9) at various time-points following lung injury, or due to technical problems with data collection (*n* = 1). In case of a pneumothorax, the experiment was terminated following validation by chest X-ray images. One pneumothorax occurred during the induction of lung injury, 2 occurred in the PVV group (1 at each PEEP level), 4 in the MVV group (1 at PEEP6 and 3 at PEEP9) and 2 in the PCS group (1 at each PEEP level). Therefore, 54 rabbits were included in the final analysis (39 females, 15 males). The number of animals included in the final analysis of the eight protocol groups is indicated in Figure 1.

The body weight of the animals did not differ between the protocol groups. The baseline data obtained under PCV ventilation at PEEP 6 cmH_2_O, did not differ among the eight groups. The multi-hit model led to severe ARDS, which manifested in profound decreases in PaO_2_/FiO_2_ ratios with more severe effects in the animals ventilated with a PEEP of 6 cmH_2_O (63.0 [44.8, 81.2] mmHg vs. 112 [95.9, 128] mmHg, *p* < 0.001 for pooled data for PEEP 6 and 9 cmH_2_O, respectively).

Absolute values of the respiratory mechanical parameters at BL under PEEP of 6 cmH_2_O and at T0 under PEEP of 6 or 9 cmH_2_O are presented in Table 1. Although no significant differences were observed between the groups at BL, increasing PEEP at T0 resulted in significantly lower values in G in the MVV and PCV groups (*p* < 0.05) and H in the PCV group (*p* = 0.02). Lung injury elevated Raw in all groups at a PEEP level of 6 cmH_2_O and in the PCV and PCS groups at a PEEP level of 9 cmH_2_O (*p* < 0.044). Moreover, G and H increased approximately four-fold in all groups (*p* < 0.01 for G and H, respectively).

**TABLE 1:** Respiratory mechanical parameters obtained at baseline (BL) and following lung injury (T0) in the rabbits ventilated with physiological variable ventilation (PVV), mathematically variable ventilation (MVV), pressure-controlled mode (PCV), and pressure controlled mode with regular sighs (PCS).

			**MP groups**					**HP groups**			
			**PVV**	**MVV**	**PCV**	**PCS**		**PVV**	**MVV**	**PCV**	**PCS**
Raw (cmH_2_O.s/l)	BL	PEEP 6	9.2 (7.3-11.1)	8.6 (6.7-10.5)	8.4 (7.5-9.2)	9.2 (6.0-12.3)	PEEP 6	9.2 (8.2-10.3)	8.7 (7.1-10.3)	8.3 (7.4-9.3)	8.7 (7.2-10.2)
	T0	PEEP 6	14.5^*^ (12.6-16.4)	12.3^*^ (9.7-14.8)	12.0^*^ (9.5-14.5)	12.0^*^ (8.3-15.6)	PEEP 9	11.1 (8.2-14.0)	10.4 (8.4-12.4)	10.7^*^ (9.9-11.6)	11.5^*^ (9.3-13.8)
G (cmH_2_O/l)	BL	PEEP 6	80.2 (67.4-93.1)	87.3 (77.0-97.5)	91.0 (76.2-105.8)	82.3 (65.0-99.6)	PEEP 6	76.8 (63.6-90.0)	86.2 (73.4-99.1)	88.9 (74.9-102.8)	91.1 (78.9-103.3)
	T0	PEEP 6	251^*^ (153-350)	309^*^ (192-426)	348^*^ (170-527)	262^*^ (142-383)	PEEP 9	177^*^ (128-226)	207^*#^ (144-269)	174^*#^ (123-225)	196^*^ (146-247)
H (cmH_2_O/l)	BL	PEEP 6	252 (220-286)	269 (233-305)	267 (235-299)	257 (243-271)	PEEP 6	246 (230-262)	255 (217-292)	274 (237-311)	277 (247-306)
	T0	PEEP 6	1105^*^ (594-1616)	1162^*^ (629-1695)	1438^*^ (817-2059)	1149^*^ (513-1785)	PEEP 9	856^*^ (453-1259)	790^*^ (504-1076)	875^*#^ (649-1101)	948^*^ (607-1289)

Raw, airway resistance; G, tissue damping; H, tissue elastance. Values displayed as group mean (95% confidence interval). ^*^p < 0.05 vs. BL, ^#^*p* < 0.05 vs. PEEP 6.

**FIGURE 2:**
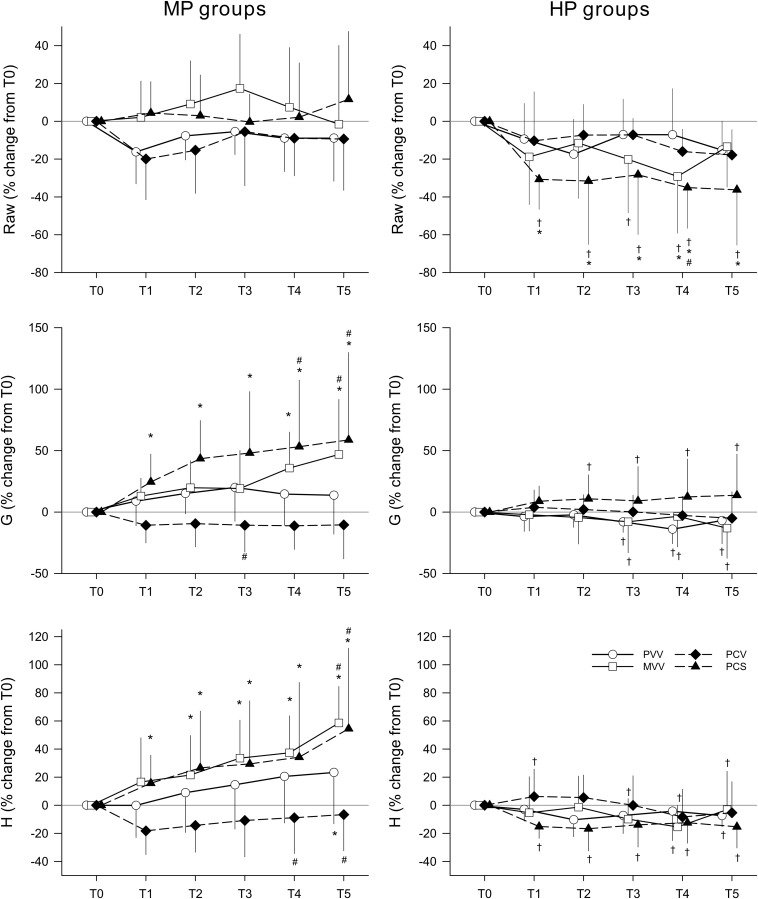
Changes in respiratory mechanical parameters during the application of various ventilation modes compared to values obtained before application (T0). Left panels display changes in groups ventilated with a PEEP of 6 cmH_2_O, whereas groups depicted in the right panels were ventilated with a PEEP of 9 cmH_2_O. Data are presented as group means ± half-width of 95% confidence interval. Raw, airway resistance; G, tissue damping; H, tissue elastance; T1–T5, measurement points at the end of each 5 h of ventilation with an experimental pattern; MP, moderate PEEP; HP, high PEEP; PVV, physiological variable ventilation; MVV, mathematical variable ventilation; PCV, pressure-controlled ventilation; PCS, pressure-controlled ventilation with regular sighs. Results of the ANOVA analyses (*p*-values) for factor “ventilation mode.” *p* = 0.44 for Raw, *p* < 0.01 for G and *p* = 0.02 for H; for factor “PEEP.” *p* < 0.01 for all variables; for the interaction of “ventilation mode ^*^PEEP *p* < 0.01 for all variables; for interaction of “ventilation mode ^*^ PEEP ^*^ time.” *p* < 0.01 for all variables. ^*^p < 0.05 vs. T0. ^#^*p* < 0.05 vs. PVV. ^†^*p* < 0.05 vs. MP.

The relative changes in the respiratory mechanical parameters compared to their corresponding values at T0 are depicted in Figure 2. In the groups ventilated with a PEEP of 6 cmH_2_O, no significant temporal changes or between-group differences in Raw were observed. Applying a PEEP level of 9 cmH_2_O led to a significant decrease in Raw in the PCS group when compared to both T0 (*p* < 0.01, T1–T5) and to the same ventilation pattern at PEEP 6 cmH_2_O (*p* < 0.02, T1–T5). The ventilation pattern affected the temporal changes in the respiratory tissue mechanical parameters (G and H) significantly when ventilating with PEEP 6 cmH_2_O. No deteriorations in G and H were observed in the PCV and PVV groups. G significantly increased both in the PCS (*p* < 0.05, T1–T5) and MVV group (*p* < 0.05, T4–T5) along with similar elevations of H in both the PCS (*p* < 0.05, T1–T5) and MVV group (*p* < 0.05, T2–T5). Values of G and H were also significantly higher in these two groups than in the PVV group (*p* < 0.05, T4–T5). Conversely, the ventilation pattern had no effect on the respiratory tissue parameters with higher PEEP, preventing the increases observed with a moderate PEEP of 6 cmH_2_O in both G (*p* < 0.05, HP vs. MP, T3–T5 in PVV, MVV, and PCS groups) and H (*p* < 0.05, HP vs. MP, groups PVV, MVV, and PCS).

**FIGURE 3:**
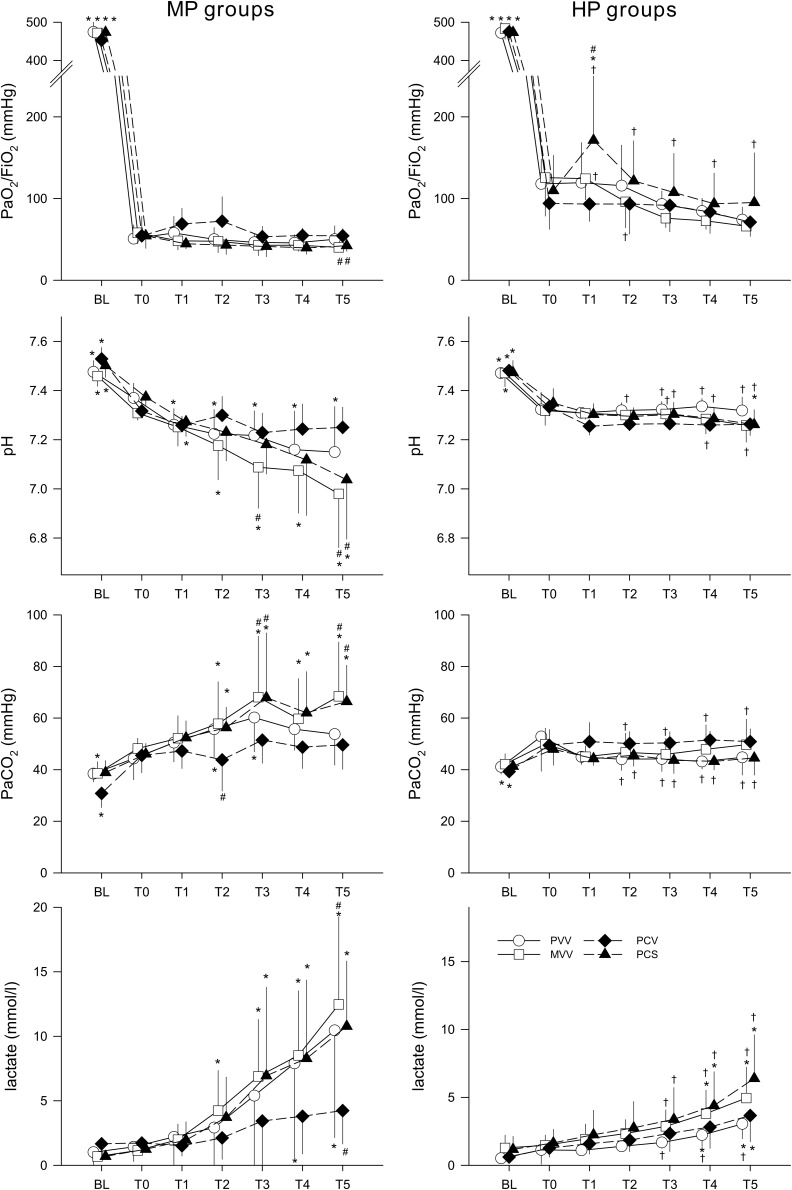
Changes in blood gas parameters before (BL), after the induction of lung injury (T0) and during the application of various ventilation modes. Left panels display changes in groups ventilated with a PEEP of 6 cmH_2_O, whereas groups depicted in the right panels were ventilated with a PEEP of 9 cmH_2_O. Data are presented as group means ± half-width of 95% confidence interval. PaO_2_, arterial partial oxygen pressure. FiO_2_, fraction of inspired oxygen. PaCO_2_, arterial partial carbon-dioxide pressure. T1–T5, measurement points at the end of each 5 h of ventilation with an experimental pattern. MP, moderate PEEP. HP, high PEEP. PVV, physiological variable ventilation. MVV, mathematical variable ventilation. PCV, pressure-controlled ventilation. PCS, pressure-controlled ventilation with regular sighs. Results of the ANOVA analyses (*p*-values) for factor “ventilation mode.” *p* < 0.01 for pH, lactate and PaCO_2_, *p* = 0.12 for PaO_2_/FiO_2_; for factor “PEEP.” *p* < 0.01 for all variables; for the interaction of “ventilation mode ^*^ PEEP”: *p* < 0.01 for pH, PaCO_2_, and PaO_2_/FiO2, *p* = 0.25 for lactate; for interaction of “ventilation mode ^*^ PEEP ^*^ time”: *p* < 0.01 for all variables. ^*^p < 0.05 vs. T0. ^#^*p* < 0.05 vs. PVV. ^†^*p* < 0.05 vs. MP.

Changes in arterial blood gas parameters are presented in Figure 3. The PaO_2_/FiO_2_ ratio was significantly lower after 5 h of ventilation in the MVV and PCS groups compared to the PVV group (*p* < 0.03, T5). Application of a higher PEEP level resulted in a significantly higher oxygenation index in the PCS group (*p* < 0.02, T1–T5). PEEP and ventilation patterns had an impact on the acid–base status. Under MP, severe acidosis was observed in the MVV and PCS groups at T5 (*p* < 0.02), whereas a smaller decrease in pH was observed in the PVV group (*p* < 0.05, T1–T5). Conversely, there was no further deterioration in the acid–base status of animals ventilated with HP: pH values were higher than those observed under MP for the PVV, MVV, and PCS groups (*p* < 0.01, T3–T5). Hypercapnia, reflecting alteration in alveolar ventilation, occurred in the MVV and PCS groups at MP (*p* < 0.05, T2–T5), with significantly higher values than the PVV group (*p* < 0.05, T3 and T5). All groups of animals ventilated with HP exhibited normocapnia with no further temporal changes, and with values significantly lower than those observed under MP (*p* < 0.04, T2–T5 in groups PVV, MVV, and PCS). At both PEEP levels, lactate increased in all groups after 3 h ventilation (*p* < 0.01, T3–T5), with values lower with HP than with MP.

**FIGURE 4:**
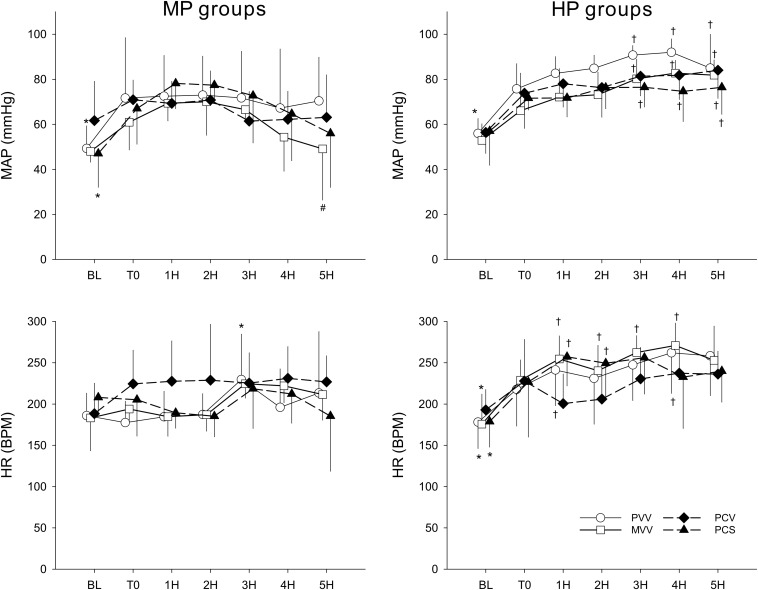
Changes in haemodynamic parameters before (BL), after the induction of lung injury (T0), and during the application of various ventilation modes. Left panels display changes in groups ventilated with a PEEP of 6 cmH_2_O, whereas groups depicted in the right panels were ventilated with a PEEP of 9 cmH_2_O. Data are presented as group means ± half-width of 95% confidence interval. MP, moderate PEEP. HP, high PEEP. MAP, mean arterial pressure. HR, heart rate. BPM, beats per minute. 1H–5H, averages of measured parameters during the corresponding 1 h period of ventilation with an experimental pattern. PVV, physiological variable ventilation. MVV, mathematical variable ventilation. PCV, pressure-controlled ventilation. PCS, pressure-controlled ventilation with regular sighs. Results of the ANOVA analyses (*p*-values) for factor “ventilation mode.” *p* < 0.01 for MAP and *p* = 0.79 for HR; for factor “PEEP”: *p* < 0.01 for all variables; for the interaction of “ventilation mode ^*^ PEEP”: *p* = 0.24 for MAP, *p* < 0.01 for HR; for interaction of “ventilation mode ^*^ PEEP ^*^ time”: *p* < 0.01 for all variables. ^*^p < 0.05 vs. T0. ^#^*p* < 0.05 vs. PVV. ^†^*p* < 0.05 vs. MP.

Changes in MAP and HR are depicted in Figure 4. The results show haemodynamic stability throughout the ventilation period in all groups of rabbits. Applying PEEP at a level of 9 cmH_2_O did not result in deterioration of blood pressure: in contrast, we observed higher values of MAP in all experimental groups (*p* < 0.04, 3H–5H). Moreover, there was a tendency for a higher HR in all groups except PCV.

Results of the histology analyses are shown in Table 2. The degree of severity of ARDS is confirmed by the high lung injury scores and the low indices reflecting alveolar aeration. There was no evidence for a difference between the experimental groups in the histology parameters regardless of the ventilation mode or the level of PEEP.

**TABLE 2:** Histology parameters obtained in the rabbits ventilated with physiological variable ventilation (PVV), mathematically variable ventilation (MVV), pressure-controlled mode (PCV), and pressure controlled mode with regular sighs (PCS) at positive expiratory pressure (PEEP) levels of 6 and 9 cmH_2_O.

	**MP groups**				**HP groups**			
	**PVV**	**MVV**	**PCV**	**PCS**	**PVV**	**MVV**	**PCV**	**PCS**
Lung injury score	0.87 (0.076)	0.88 (0.072)	0.83 (0.072)	0.88 (0.171)	0.89 (0.069)	0.87 (0.034)	0.86 (0.084)	0.87 (0.042)
Global aeration score	0.67 (0.063)	0.67 (0.042)	0.65 (0.068)	0.67 (0.000)	0.65 (0.021)	0.65 (0.021)	0.67 (0.031)	0.65 (0.021)
Average aeration per FOV	0.65 (0.060)	0.63 (0.031)	0.64 (0.064)	0.63 (0.047)	0.65 (0.016)	0.65 (0.021)	0.65 (0.036)	0.63 (0.029)
Hemorrhage score	1.67 (0.66)	1.75 (0.47)	1.15 (0.29)	1.34 (0.42)	1.27 (0.28)	1.44 (0.50)	1.22 (0.155)	1.16 (0.234)
Alveolar septal thickening (oedema) score	0.61 (0.73)	0.71 (0.31)	0.40 (0.25)	0.56 (0.63)	0.68 (0.17)	0.75 (0.31)	0.35 (0.48)	0.50 (0.30)
Wet-to-dry lung weight ratio	10.03 (4.67)	11.15 (1.49)	8.87 (1.82)	10.54 (1.97)	10.09 (1.00)	9.92 (0.69)	10.12 (1.38)	10.07 (1.38)

*Values displayed as group median (interquartile range). No statistically significant differences were observed. FOV, field of view; Lung injury score is a continuous variable interpreted between 0 (no injury) and 1 (severe injury). Aeration scores categorized as 1/3, 1/2, or 2/3. Hemorrhage scores classified as 0 (none), 1 (light), 2 (moderate), or 3 (severe). Alveolar septal thickening score is classified as 0 (<2 per FOV), 1 (2–4 per FOV), or 2 (>4 per FOV).*

**FIGURE 5:**
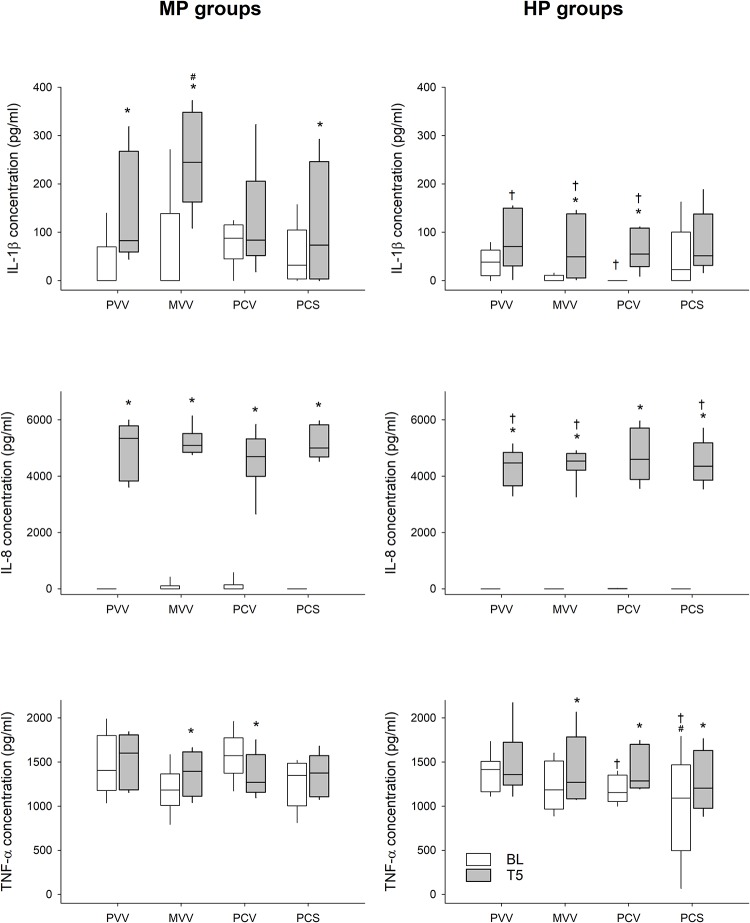
Changes in inflammatory cytokine levels during the application of various ventilation modes compared to values obtained at the beginning of the experiment (BL). Left panels display changes in groups ventilated with a PEEP of 6 cmH_2_O, whereas groups depicted in the right panels were ventilated with a PEEP of 9 cmH_2_O. PVV, physiological variable ventilation. MVV, mathematical variable ventilation. PCV, pressure-controlled ventilation. PCS, pressure-controlled ventilation with regular sighs. Results of the ANOVA analyses (*p*-values) for factor “ventilation mode”: *p* = 0.35 for IL-1β, *p* = 0.14 for IL-8, *p* < 0.01 for TNF-α; for factor “PEEP”: *p* < 0.01 for all variables; for the interaction of “ventilation mode ^*^ PEEP”: *p* = 0.01 for IL-1β, *p* = 0.18 for IL-8 and TNF-α; for interaction of “ventilation mode ^*^ PEEP ^*^ time”: *p* < 0.01 for IL-1β, *p* = 0.04 for IL-8 and TNF-α. ^*^p < 0.05 vs. BL. ^#^*p* < 0.05 vs. PVV. ^†^*p* < 0.05 vs. MP.

Changes in serum cytokine levels are provided in Figure 5. No differences were found in the baseline cytokine serum levels between any of the groups, regardless of the PEEP allocation. Both IL-1β and IL-8 exhibited a significant increase in all groups at both PEEP levels following lung injury. A different response was observed for TNF-α: concentrations were the same at both PEEP levels in the PVV group, whereas a significant increase was observed in all other groups compared to baseline.

## Discussion

We compared changes in respiratory mechanics and lung function following prolonged ventilation with monotonous or variable ventilation modalities in an experimental model of ARDS, in which moderate or high PEEP levels were applied. At moderate PEEP, mechanical ventilation with regular sighs was as detrimental to lung function as a MVV. Applying variable ventilation based on a pre-recorded physiological breathing pattern or conventional pressure-controlled ventilation proved to be less detrimental for respiratory mechanics and gas exchange parameters than the application of a mathematically derived variable pattern over a 5-h period. Elevating PEEP protected against the deleterious effects of the 5-h mechanical ventilation, regardless of the ventilation mode.

The respiratory mechanical parameters obtained as the main outcomes in the present study are in line with the baseline values at PEEP 6 cmH_2_O that were previously obtained in the same species (Albu et al., 2013). Furthermore, changes in the mechanical parameters with a predominant increase in the respiratory tissue parameters are in qualitative accordance with earlier findings using similar model of ARDS (Bellardine et al., 2006; Guldner et al., 2018). However, the respiratory mechanical changes were more severe in the present study than those observed previously (Mutch et al., 2000; Funk et al., 2004; Bellardine et al., 2006; Guldner et al., 2018), and this was also reflected in the severe deterioration of gas exchange (Figure 3). These exaggerated effects may be attributed to the added effect of lung inflammation induced by intravenous LPS in the present injury model. These findings suggest that the increases in G reflect severely compromised lung tissue damping in the pulmonary parenchyma, loss of lung volume and/or enhanced ventilation heterogeneities (Lutchen et al., 1996; Albu et al., 2013). The marked elevations in H are the result of lung tissue stiffening subsequent to surfactant depletion, development of interstitial and intra-alveolar edema and/or the loss of ventilated alveolar units (Albu et al., 2013; Bayat et al., 2013).

Recently, there has been growing interest for introducing variability in V_T_ to improve alveolar recruitment and lung aeration, both of which deteriorate with time under monotonous conventional ventilation (Muscedere et al., 1994; Evans et al., 2014; Bates and Smith, 2018). Although many experimental studies have demonstrated some protective effect of variable ventilation on the progression of lung injury (Mutch et al., 2000; Kiss et al., 2016; Guldner et al., 2018), the results of the present study demonstrate that the choice of the distribution of V_T_ plays a major role in the ability to prevent progressive deterioration of lung function. Applying a previously established mathematically derived waveform (Thammanomai et al., 2008) in mechanical ventilation (Group MVV) involved more frequent delivery of excessively high V_T_, leading to higher driving pressure (Supplementary Figure S3 in data supplement). Since the driving pressure is primarily responsible for lung tissue strain (Protti et al., 2015; Chiumello et al., 2016), it is not surprising that the MVV and PCS ventilation modes were the least optimal under MP. These modes gradually further deteriorated lung function (Figure 2 left).

The progression of lung injury observed in the present study with MVV is in contrast with earlier results demonstrating either a protective effect with stabilization of lung injury (Thammanomai et al., 2008, 2013) or an improvement (Bartolak-Suki et al., 2017). This discrepancy may be attributed to the underlying pathophysiological mechanism of ARDS. In the present study, we mimic a clinical condition combining three key mechanisms that contribute to ARDS with concomitant induction of inflammation (LPS), surfactant depletion (lavage) and shear stress (high-V_T_ injurious ventilation at ZEEP). The resulting degree of induced lung injury in the present study was severe according to the Berlin definition (ARDS Definition Task Force et al., 2012) which was also apparent in the high values of the histological lung injury scores (Table 2). Previous studies characterized the effects of ventilation modes on the progression of ARDS in models using single mechanisms opposed to the multi-hit approach of the present study, focusing only on surfactant damage (Bellardine et al., 2006; Spieth et al., 2009; Guldner et al., 2018) or damage to the alveolar-capillary barrier with oleic acid (Mutch et al., 2000; Boker et al., 2002; Funk et al., 2004). Considering that the degree of lung tissue inflammation varies between the different experimental models, the discrepancy observed between studies in which the mathematically derived variable ventilation pattern was applied may be due to differences in the ARDS phenotype (Laffey and Kavanagh, 2018).

Elevating PEEP recruited closed alveolar units as reflected by the lower G and H values (Table 1). The significantly lowered cytokine levels in the HP groups also suggest decreased inflammation, likely as a result of less alveolar shear stress (Figure 5), leading to better lung aeration and reduced atelectrauma. This beneficial effect of PEEP appears to be more effective than the ventilation mode *per se* in terms of respiratory mechanics, gas exchange and inflammatory cytokine concentrations (Figures 2, 3, 5 right).

The lack of progression in the severity of ARDS during the 5-h mechanical ventilation using a conventional PCV mode with low V_T_ is in agreement with the protective ventilation strategy concept (Sahetya et al., 2017; Fan et al., 2018). Interestingly, PVV was able to provide essentially the same benefit both on lung function and on gas exchange (Figures 2, 3). This beneficial effect of the pre-recorded physiological breathing pattern in severe ARDS is in agreement with recent findings in healthy lungs (Walesa et al., 2018). However, although this beneficial effect was also apparent in the inflammatory cytokine levels, it was not observed for the histological findings, which revealed no differences between the different ventilation patterns and PEEP (Table 2). Since the lack of histological difference was also reported previously in the presence of severe ARDS (Guldner et al., 2018), the severity of ARDS and/or the time lag between the initiation of the ventilation mode and the histological changes may explain the discrepancies between the functional and structural findings. On one hand, the lung injury scores were at the upper limit of severity, and on the other hand, there may not have been enough time to observe a difference in the lung histology between groups. Further studies using longer time frames are needed to address the impact of ventilation modes on lung morphology.

A few methodological aspects relevant to the study design should be addressed. A number of mathematically derived variable ventilation patterns have been investigated previously. These patterns relied on different statistical distributions of V_T_, such as Gaussian (Kiss et al., 2016; Wierzchon et al., 2017; Camilo et al., 2018; Guldner et al., 2018; Spieth et al., 2018), white noise (Froehlich et al., 2008; Guldner et al., 2018), or skewed (Thammanomai et al., 2008; Bartolak-Suki et al., 2017). In the present study, we decided to apply the latter as this particular pattern was designed specifically to optimize lung recruitment in a murine model of acute lung injury (Thammanomai et al., 2008). While the application of a breathing pattern obtained in animals with lung injury as a PVV pattern might also allow investigation of physiological adaptation mechanisms, we chose to apply breathing patterns obtained in healthy animals since this particular pattern was demonstrated to have beneficial effects in an earlier study (Walesa et al., 2018). Previous studies comparing differences between white noise derived and PVV patterns found no difference in respiratory parameters in the presence of oleic acid lung injury (Froehlich et al., 2008). This finding might be related to the similarity of the two ventilation patterns used in this earlier study, as opposed to the much greater difference between the two variable patterns applied in our study in terms of V_T_ distribution.

Further methodological aspects potentially affecting the experimental outcomes may be related to the severity of the systemic inflammation apparent from the significant elevations in lactate levels. However, all animals received comparable amounts of i.v. crystalloids. In addition, the histological oedema scores and the wet/dry lung weight ratio did not differ among the groups.

The selection of the animal model might also warrant some methodological considerations. While previous studies investigating variable mechanical ventilation in animals used large mammals, such as pigs or sheep (Mutch et al., 2000; Boker et al., 2002; Funk et al., 2004; Bellardine et al., 2006; Spieth et al., 2009) or small rodents, i.e., mice or rats (Thammanomai et al., 2008, 2013; Kiss et al., 2016; Camilo et al., 2018), our protocol utilized a rabbit model. There are widely accepted differences in tidal volume and respiratory rate, which are mainly attributed to be effects of body size (Bide et al., 2000). Furthermore, some structural and functional differences of the respiratory system exist between these species (Negus, 1955; Patra, 1986; Gomes et al., 2000), nevertheless, the fundamental physiological and pathological processes are universal among these mammals, and the mechanical properties of the respiratory system scale proportionally to size (Fredberg and Stamenovic, 1989; Gomes et al., 2000), allowing the comparison of results obtained in different models.

To overcome the multiple comparisons problem and to assess the validity of the use of multiple three-way repeated ANOVA analyses, a principle component analysis was performed. The primary experimental outcome variables, i.e., respiratory mechanical variables Raw, G and H along with blood gas parameters PaO_2_/FiO_2_, PaCO_2_, arterial pH and lactate were incorporated in this analysis by calculating the relative changes at T5 in each variable compared to T0 (calculated as T5/T0 – 1). Results of the principle component analysis are presented in the online data supplement (Supplementary Tables S1, S2 and Supplementary Figure S7, with Supplementary Table S1 containing the weights for the newly identified components. The three main components (PC1, PC2, and PC3) identified in this analysis explain 88% of the total variation (Supplementary Table S2). PC1 was mainly determined by G, H, PaCO_2_, lactate and pH with approximately equal weights, PC2 was mainly determined by Raw and PC3 mainly consisted of PaO_2_/FiO_2_. Two-way ANOVA analyses with factors PEEP and ventilation mode were carried out on these three components, yielding similar trends to those observed in the individual variables, validating our statistical analyses.

## Conclusion

In summary, changes in respiratory mechanical, gas exchange and histological parameters were compared in rabbits with severe ARDS ventilated either with conventional monotonous or variable ventilation patterns. With a moderate PEEP (6 cmH_2_O), both a variable ventilation based on pre-recorded physiological breathing pattern (PVV) and the conventional pressure control mode (PCV) prevented further lung function deterioration that was otherwise observed in the animals ventilated with PCS and MVV patterns. The use of PCV was also associated with a further benefit on tissue mechanics compared to PVV. Applying an elevated PEEP of 9 cmH_2_O prevented lung injury progression even when the more deleterious ventilation modes were utilized. These findings indicate that while elevation of PEEP is essential in a protective ventilation strategy to keep the lung open, PVV can be a valid synergistic alternative to the conventional protective approach of PCV to avoid progression of lung injury, including when elevating PEEP may jeopardize cardiac output.

## Data Availability

The datasets generated for this study are available on request to the corresponding author.

## Ethics Statement

The experimental protocol was approved by the Experimental Ethics Committee of the University of Geneva and the Animal Welfare Committee of the Canton of Geneva, Switzerland (No. GE/94/15, 27 August 2015). All procedures were performed according to the current animal protection laws of Switzerland (LPA, RS455) and reported in compliance with ARRIVE guidelines.

## Author Contributions

GF: study design, data collection and analysis, manuscript drafting. SB and FP: study design, data analysis and interpretation, manuscript drafting. GA: study design, data collection and analysis. NL: data collection and analysis. AB: data collection and histology analysis. JD: data collection and immunology analysis. WH: study design, data interpretation, manuscript drafting.

## Conflict of Interest Statement

Engineering support was provided by Getinge AB (Solna, Sweden) by providing special firmware for the ventilator that allowed the application of variable ventilation patterns. The authors declare that the research was conducted in the absence of any commercial or financial relationships that could be construed as a potential conflict of interest.
