# Supplementary material for: Variable Ventilation Is Equally Effective as Conventional Pressure Control Ventilation for Optimizing Lung Function in a Rabbit Model of ARDS
Source: Front Physiol. 2019 Jun 26;10:803. doi: 10.3389/fphys.2019.00803 (PMC6607923; doi:10.3389/fphys.2019.00803)
Supplement: Supplementary file 1 [file Table_1.docx]

**Online data supplement**

**variable ventilation is equalLY EFFECTIVE AS conventional pressure control ventilation for** **optimizing lung function in a rabbit model of ards**

Gergely H. Fodor^1^, Sam Bayat^1,2^, Gergely Albu^1^, Na Lin^1,3^, Aurélie Baudat^1^, Judit Danis^4^, Ferenc Petak^5^, Walid Habre^1^

^1^ Unit for Anaesthesiological Investigations, University Hospitals of Geneva and University of Geneva, 6, rue Willy Donzé, 1205 Geneva, Switzerland

^2^ Inserm UA7 STROBE Laboratory & Department of Clinical Physiology, Sleep and Exercise, Grenoble University Hospital, Boulevard de la Chantourne, 38700 La Tronche, Grenoble, France

^3^Department of Anesthesiology, Beijing Tongren Hospital, Capital Medical University, 1# Dongjiaominxiang Street, 100730 Beijing, China,

^4^MTA-SZTE Dermatological Research Group, University of Szeged, 6 Koranyi fasor, 6720 Szeged, Hungary

^5^ Department of Medical Physics and Informatics, University of Szeged, 9 Koranyi fasor, 6720 Szeged, Hungary

**Figure S1:** Recordings used to control the pressure delivered by the ventilator during the application of the different ventilation modes. For the variable patterns (top two panels), the displayed data represent one time period, which was repeatedly applied for 5 hours, whereas for pressure-control modes (bottom two panels), representative sections are displayed.

PVV: physiological variable ventilation. MVV: mathematical variable ventilation. PCV: pressure-controlled ventilation. PCS: pressure-controlled ventilation with regular sighs.

**Figure S2:** Changes in tidal volume normalized to body weight before (BL), after the induction of lung injury (T0) and during the application of various ventilation modes. Left panels display changes in groups ventilated with a PEEP of 6 cmH_2_O, whereas groups depicted in the right panels were ventilated with a PEEP of 9 cmH_2_O. Depicted are the median values (top), the minima (middle) and the maxima obtained during each analysis period (bottom). Data are presented as group means ± half-width of 95% confidence interval.

V_T_e: expired tidal volume. 1H–5H: averages of measured parameters during the corresponding 1 hour period of the ventilation with an experimental pattern. BL and T0 were calculated during 30 second intervals, whereas 1H–5H were calculated over 1 hour periods. MP: moderate PEEP. HP: high PEEP. PVV: physiological variable ventilation. MVV: mathematical variable ventilation. PCV: pressure-controlled ventilation. PCS: pressure-controlled ventilation with regular sighs.

*: p < 0.05 vs. T0. #: p < 0.05 vs. PVV. †: p < 0.05 vs. MP.

**Figure S3:** Changes in ventilatory driving pressure above PEEP before (BL), after the induction of lung injury (T0) and during the application of various ventilation modes. Left panels display changes in groups ventilated with a PEEP of 6 cmH_2_O, whereas groups depicted in the right panels were ventilated with a PEEP of 9 cmH_2_O. Depicted are the median values (top), the minima (middle) and the maxima (bottom) obtained during each analysis period. Data are presented as group means ± half-width of 95% confidence interval.

1H–5H: averages of measured parameters during the corresponding 1 hour period of the ventilation with an experimental pattern. BL and T0 were calculated during 30 second intervals, whereas 1H–5H were calculated over 1 hour periods. MP: moderate PEEP. HP: high PEEP. PVV: physiological variable ventilation. MVV: mathematical variable ventilation. PCV: pressure-controlled ventilation. PCS: pressure-controlled ventilation with regular sighs.

*: p < 0.05 vs. T0. #: p < 0.05 vs. PVV. †: p < 0.05 vs. MP.

**Figure S4:** Changes in respiratory rate before (BL), after the induction of lung injury (T0) and during the application of various ventilation modes. Left panels display changes in groups ventilated with a PEEP of 6 cmH_2_O, whereas groups depicted in the right panels were ventilated with a PEEP of 9 cmH_2_O. Depicted are the median values (top), the minima (middle) and the maxima (bottom) obtained during each analysis period. Data are presented as group means ± half-width of 95% confidence interval.

RR: respiratory rate. BPM: breaths per minute. 1H–5H: averages of measured parameters during the corresponding 1 hour period of the ventilation with an experimental pattern.

BL and T0 were calculated during 30 second intervals, whereas 1H–5H were calculated over 1 hour periods. MP: moderate PEEP. HP: high PEEP. PVV: physiological variable ventilation. MVV: mathematical variable ventilation. PCV: pressure-controlled ventilation. PCS: pressure-controlled ventilation with regular sighs.

*: p < 0.05 vs. T0. #: p < 0.05 vs. PVV. †: p < 0.05 vs. MP.

**Figure S5:** Changes in variability indices of respiratory rate, driving pressure and normalized tidal volumes before (BL), after the induction of lung injury (T0) and during the application of various ventilation modes. Left panels display changes in groups ventilated with a PEEP of 6 cmH_2_O, whereas groups depicted in the right panels were ventilated with a PEEP of 9 cmH_2_O. Depicted are the median values (top), the minima (middle) and the maxima (bottom) obtained during each analysis period. Data are presented as group means ± half-width of 95% confidence interval.

V_T_e: expired tidal volume. RR: respiratory rate. BPM: breaths per minute.

1H–5H: averages of measured parameters during the corresponding 1 hour period of the ventilation with an experimental pattern. BL and T0 were calculated during 30 second intervals, whereas 1H–5H were calculated over 1 hour periods.

MP: moderate PEEP. HP: high PEEP. PVV: physiological variable ventilation. MVV: mathematical variable ventilation. PCV: pressure-controlled ventilation. PCS: pressure-controlled ventilation with regular sighs.

*: p < 0.05 vs. T0. #: p < 0.05 vs. PVV. †: p < 0.05 vs. MP.

**Figure S6:** Number of breaths with a normalized tidal volume greater or equal to 10 ml/kg delivered and changes of normalized minute ventilation before (BL), after the induction of lung injury (T0) and during the application of various ventilation modes. Left panels display changes in groups ventilated with a PEEP of 6 cmH_2_O, whereas groups depicted in the right panels were ventilated with a PEEP of 9 cmH_2_O. Depicted are the median values (top), the minima (middle) and the maxima (bottom) obtained during each analysis period. Data are presented as group means ± half-width of 95% confidence interval.

V_T_e: expired tidal volume. 1H–5H: averages of measured parameters during the corresponding 1 hour period of the ventilation with an experimental pattern.

BL and T0 were calculated during 30 second intervals, whereas 1H–5H were calculated over 1 hour periods.

MP: moderate PEEP. HP: high PEEP. PVV: physiological variable ventilation. MVV: mathematical variable ventilation. PCV: pressure-controlled ventilation. PCS: pressure-controlled ventilation with regular sighs.

*: p < 0.05 vs. T0. #: p < 0.05 vs. PVV. †: p < 0.05 vs. MP.

|  | **PC1** | **PC2** | **PC3** | **PC4** | **PC5** | **PC6** | **PC7** |
| --- | --- | --- | --- | --- | --- | --- | --- |
| **G** | -0.394 | 0.450 | -0.273 | 0.045 | -0.239 | 0.692 | -0.175 |
| **H** | -0.448 | 0.149 | -0.122 | 0.402 | -0.477 | -0.506 | 0.342 |
| **Raw** | -0.202 | -0.796 | 0.197 | 0.283 | -0.276 | 0.359 | -0.034 |
| **arterial pH** | 0.465 | -0.026 | -0.200 | -0.206 | -0.351 | 0.274 | 0.709 |
| **PaO_2_/FiO_2_** | 0.036 | 0.333 | 0.905 | -0.013 | -0.224 | 0.107 | 0.088 |
| **lactate** | -0.416 | -0.171 | 0.028 | -0.845 | -0.243 | -0.152 | -0.032 |
| **PaCO_2_** | -0.461 | -0.027 | 0.110 | 0.000 | 0.638 | 0.166 | 0.584 |

**Table S1.** Resulting weights of the principle component analysis. G: tissue damping, H: tissue elastance, Raw: airway resistance, PaO_2_: arterial partial pressure of oxygen, FiO_2_: fraction of inspired oxygen, PaCO_2_: arterial partial pressure of carbon-dioxide. PC1-7: the newly identified principle components.

|  | **PC1** | **PC2** | **PC3** | **PC4** | **PC5** | **PC6** | **PC7** |
| --- | --- | --- | --- | --- | --- | --- | --- |
| **Standard deviation** | 1.993 | 1.075 | 1.019 | 0.602 | 0.531 | 0.332 | 0.280 |
| **Proportion of variance** | 0.567 | 0.165 | 0.149 | 0.052 | 0.040 | 0.016 | 0.011 |
| **Cumulative proportion** | 0.567 | 0.733 | 0.881 | 0.933 | 0.973 | 0.989 | 1.000 |

**Table S2.** Importance of components for the principle component analysis. PC1-7: the newly identified principle components.


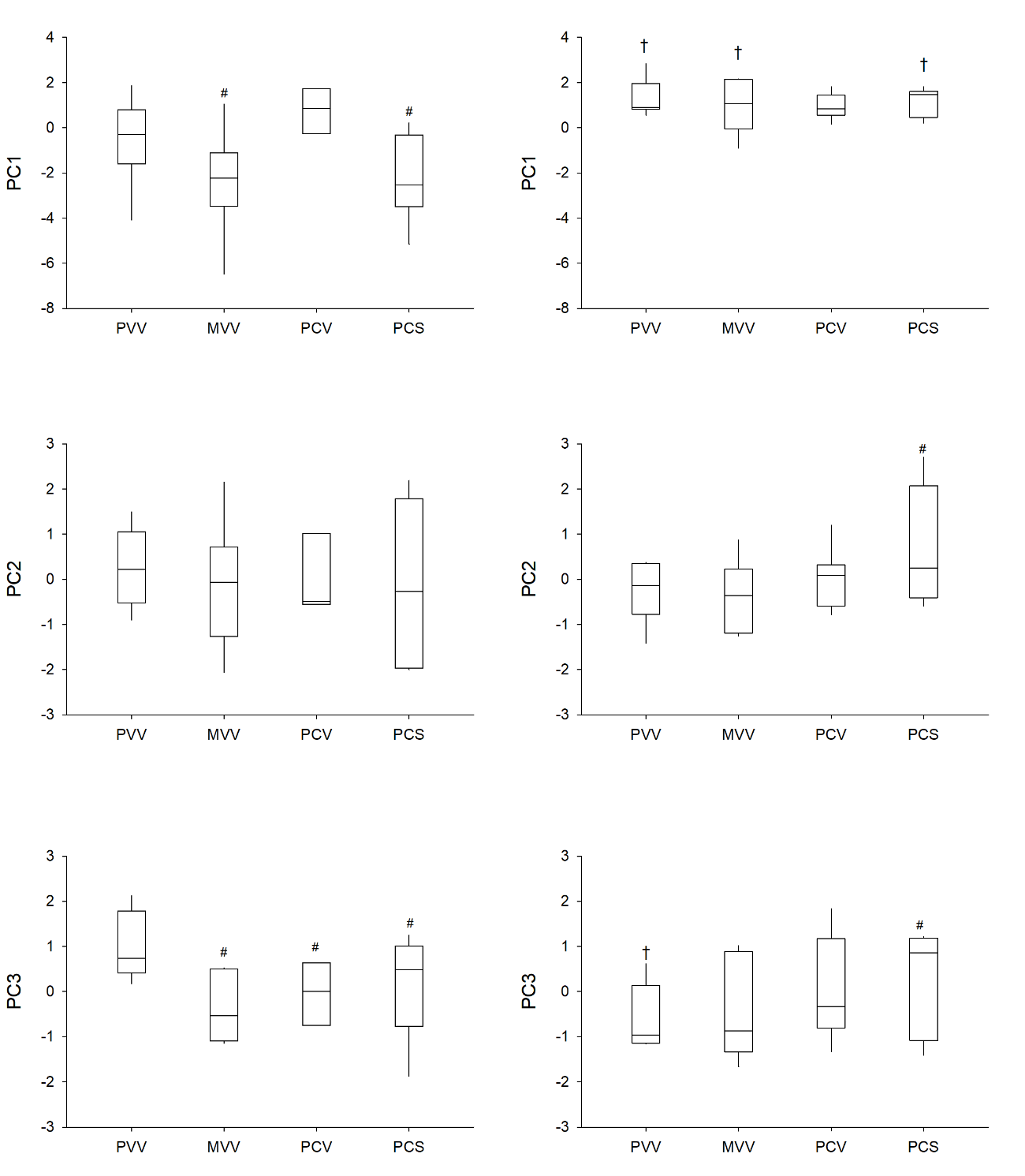


**Figure S7**: Results of the principle component analysis: changes of the three most important components (PC1-3) at the last timepoint (T5) compared to T0 calculated by the analysis using the weights presented in Table 3. Left panels display changes in groups ventilated with a PEEP of 6 cmH2O, whereas groups depicted in the right panels were ventilated with a PEEP of 9 cmH2O.

MP: moderate PEEP. HP: high PEEP. PVV: physiological variable ventilation. MVV: mathematical variable ventilation. PCV: pressure-controlled ventilation. PCS: pressure-controlled ventilation with regular sighs.

Results of the ANOVA analyses (p-values) for factor “ventilation mode”: p = 0.01 for PC1, p = 0.36 for PC2 and p = 0.07 for PC3; for factor “PEEP”: p < 0.01 for PC1, p = 0.98 for PC2 and p = 0.04 for PC3; for the interaction of “ventilation mode * PEEP”: p < 0.01 for PC1 and PC3, p = 0.15 for PC2.

#: p < 0.05 vs. PVV. †: p < 0.05 vs. MP.
